# Recent Topics on The Mechanisms of Immunosuppressive Therapy-Related Neurotoxicities

**DOI:** 10.3390/ijms20133210

**Published:** 2019-06-29

**Authors:** Wei Zhang, Nobuaki Egashira, Satohiro Masuda

**Affiliations:** 1 Department of Clinical Pharmacology and Biopharmaceutics, Graduate School of Pharmaceutical Sciences, Kyushu University, Fukuoka 812-8582, Japan; 2 Department of Pharmacy, Kyushu University Hospital, Fukuoka 812-8582, Japan

**Keywords:** alloimmune response, immunosuppressants, calcineurin inhibitors, corticosteroids, mTOR inhibitors, neurotoxicity, neuroprotective effects

## Abstract

Although transplantation procedures have been developed for patients with end-stage hepatic insufficiency or other diseases, allograft rejection still threatens patient health and lifespan. Over the last few decades, the emergence of immunosuppressive agents such as calcineurin inhibitors (CNIs) and mammalian target of rapamycin (mTOR) inhibitors have strikingly increased graft survival. Unfortunately, immunosuppressive agent-related neurotoxicity commonly occurs in clinical practice, with the majority of neurotoxicity cases caused by CNIs. The possible mechanisms through which CNIs cause neurotoxicity include increasing the permeability or injury of the blood–brain barrier, alterations of mitochondrial function, and alterations in the electrophysiological state. Other immunosuppressants can also induce neuropsychiatric complications. For example, mTOR inhibitors induce seizures, mycophenolate mofetil induces depression and headaches, methotrexate affects the central nervous system, the mouse monoclonal immunoglobulin G2 antibody (used against the cluster of differentiation 3) also induces headaches, and patients using corticosteroids usually experience cognitive alteration. Therapeutic drug monitoring, individual therapy based on pharmacogenetics, and early recognition of symptoms help reduce neurotoxic events considerably. Once neurotoxicity occurs, a reduction in the drug dosage, switching to other immunosuppressants, combination therapy with drugs used to treat the neuropsychiatric manifestation, or blood purification therapy have proven to be effective against neurotoxicity. In this review, we summarize recent topics on the mechanisms of immunosuppressive drug-related neurotoxicity. In addition, information about the neuroprotective effects of several immunosuppressants is also discussed.

## 1. Introduction

The first kidney transplant, performed by Murray et al. in 1954 [1], heralded a new age for patients with terminal hepatic insufficiency, end-stage renal diseases, and other severe diseases. However, the one-year survival rate of transplant patients was only 35% in the 1960s and 1970s and did not significantly increase until the development of ciclosporin A (cyclosporine, CsA) and tacrolimus (FK506) [2]. Strikingly, the rapid development of drugs to induce and maintain immunosuppression, such as antibodies and anti-metabolic drugs, has helped to increase graft and one-year patient survival to more than 90% in recent years [3]. Based on pharmacological mechanisms, immunosuppressive agents can be divided into six categories: calcineurin inhibitors (CNIs), mammalian target of rapamycin (mTOR) inhibitors, cell cycle inhibitors, corticosteroids, monoclonal and polyclonal antibodies, and other newly developed drugs [4].

Although many benefits have been realized, postoperative complications remain unsolved and influence the quality of life and long-term survival rates of transplant patients [5]. Among all postoperative complications, neurological problems are frequent, both in the immediate operation period and for many years after transplantation; they are associated with a poor prognosis and significant morbidity [6,7]. For example, van de Beek and colleagues [8] reported that the rate of perioperative neurological complications was associated with one-year mortality and rose from 19% to 30% in the past 10 years, as shown in a retrospective cohort study. Furthermore, the risk of neurological complications was shown to be 81% in patients during 18 years of follow-up. Common complications seen with all types of transplantation include alterations of consciousness, seizures, encephalopathy, and cerebrovascular events [9,10,11,12]. The etiologies of neurological complications are diverse, including immunosuppressant-related neurotoxicity [13,14], infections [15], metabolic disorders, hemorrhages [9], and primitive diseases prior to the transplant. Neurotoxicity induced by immunosuppressive agents has remained a severe problem in clinical practice because they degrade the quality of life for patients. For example, CNIs may induce mild symptoms, such as tremors, or severe symptoms, such as seizures, central pontine myelinolysis (CPM), and cortical blindness. Treatment with a mouse monoclonal immunoglobulin G2 antibody to the cluster of differentiation 3 (muromonab-CD3, trade name: Orthoclone OKT3^®^) is associated with headaches and aseptic meningitis. These clinical features and risk factors are well understood. However, the specific mechanisms of immunosuppressant-related neurotoxicity, and its predictive factors, remain obscure.

Over the last few decades, several attempts have been made to elucidate the pathogenesis of immunosuppressant-related neurotoxicity and to recognize its heralding symptoms. In this article, we focus on the clinical features, risk factors, pathological mechanisms, and the management of neurotoxicity induced by immunosuppressive agents.

## 2. Alloimmune Response

Once cells, tissues, or organs are transplanted between a donor and a genetically non-identical recipient (allograft transplantation), many cells, including T cells, B cells, and macrophages, are activated and participate in immune events that can initiate an alloimmune response and, finally, induce allograft rejection.

### 2.1. Allorecognition

**Figure 1:**
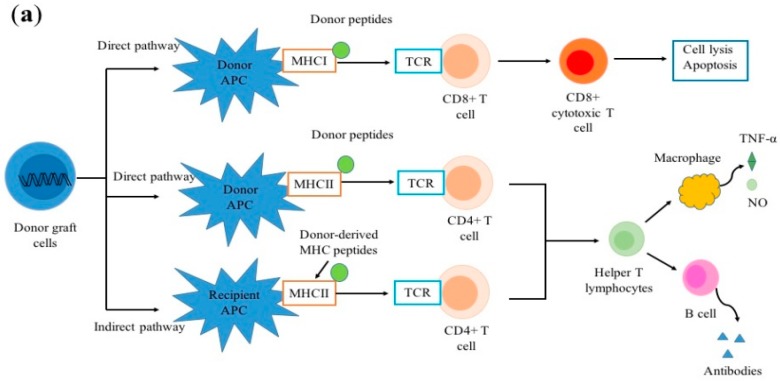

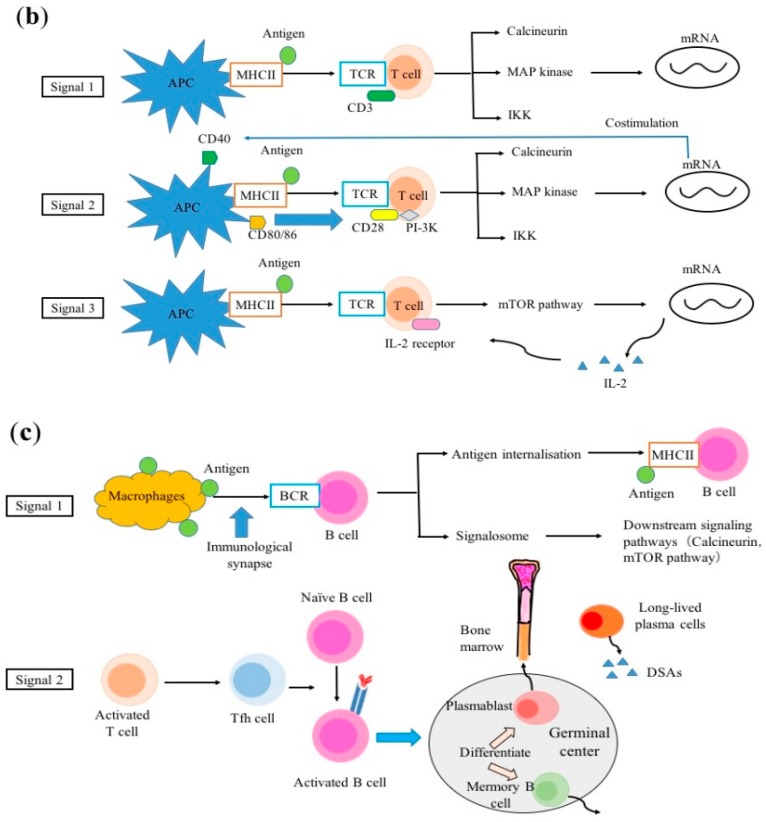
T cells, B cells, and macrophages initiate alloimmune responses and induce allograft rejection after transplantation. (**a**) Allorecognition can be initiated by direct or indirect pathways; (**b**) Three signals participate in the activation of T cells; (**c**) Two signal processes are involved in the activation of B cells. APC, antigen-presenting cell; BCR, B-cell receptor; DSA, donor specific antibody; IKK, inhibitor of NF-κB kinase; IL-2, interleukin-2; NO, nitric oxide; MAP, mitogen-activated protein; MHC, major histocompatibility complex; PI-3K, phosphatidylinositol 3-kinase; TCR, T-cell receptor; Tfh, T follicular helper; TNF-α, tumor necrosis factor alpha.

As shown in Figure 1a, allorecognition is initiated by two pathways: (1) activated T cells with direct alloreactivity interact with major histocompatibility complex (MHC) molecule–peptide complexes on donor antigen presenting cells (APCs) and induce donor cell apoptosis through cellular rejection [16], and (2) donor peptides bound to self-derived MHC molecule peptide complexes processed by recipient APCs are recognized by recipient T cells and then cause allograft destruction [17]. Nowadays, a distinct pathway, semi-direct allorecognition has been studied in the context of transplantation.

### 2.2. T-cell Activation

T cells can be activated by three types of signals (Figure 1b). First, there is the T-cell receptor-CD3 (TCR-CD3) complex on CD4+ T cells, which delivers cognate antigens and forms T-cell receptor-major histocompatibility complex allopeptides that activate a series of biochemical reactions. Second, T cells that have received an initial signal activation are activated by the interaction between CD80 or CD86 in APCs and CD28 molecules in T cells, finally generating a co-stimulatory signal, thereby initiating immunological activation. Several signaling pathways, including the calcineurin pathway, renin–angiotensin system (RAS)/mitogen activated protein (MAP) kinase pathway, and nuclear factor kappa B (NFκB), inhibitor of NFκB kinase (IKK) pathway have been reported to participate in these two activation processes. The third type of activation signal involves the binding of interleukin-2 (IL-2) with the gamma chain of its receptor to initiate T cell proliferation, DNA synthesis, and cell division through the activation of mTOR pathways [18].

### 2.3. B-cell Activation

Two signal processes account for the activation of B cells [19], as described in detail in Figure 1c. The first activation signal occurs when macrophages in the subcapsular sinus capture the cognate antigens, and then, these antigens on macrophages bind with the surface B cell receptors (BCRs) of native B cells, forming an immunological synapse [20]. Signaling BCR microclusters are involved in the process of moving antigens into the endosomal compartments of B cells and in the expression of a series of factors that play an important role in regulating downstream signaling pathways, including calcineurin and mTOR pathways [19,21]. The antigens are then processed enzymatically, internalized, and ultimately selected to present on the surface of the B cell associated with MHC II molecules [22].

Native B cells are activated with the help of follicular helper T (Tfh) cells, which initiate cell co-stimulation interactions that produce cytokines [23,24,25]. The activated B cells migrate into the germinal center where some of them differentiate into memory B cells or plasmablasts. Plasmablasts further differentiate into long-lived plasma cells on bone marrow, which can secrete high-affinity donor-specific antibodies that participate in antibody-mediated rejection [26,27].

## 3. Classification of Immunosuppressants

The functions of T and B lymphocytes in the process of rejection have become gradually understood, and the immunosuppressive regime has been optimized as a result of many experimental and clinical studies. Immunosuppressants are classified according to their mechanisms of action, as shown in Table 1 [3,4,18,28,29,30,31,32,33,34]. CNIs inhibit the activity of a calcium-dependent phosphatase named calcineurin, thereby impeding the transduction of the nuclear factor of activated T cells (NFAT) and the production of cytokines, such as IL-2, tumor necrosis factor-alpha (TNF-α), and interferon-gamma (IFN-γ). mTOR inhibitors suppress the translation of mRNA-encoding proteins, T cell proliferation, and cytokine production. Antimetabolites inhibit the synthesis of purine by diverse mechanisms, such as inhibiting inosine-5′-monophosphate dehydrogenase (IMPDH) or incorporating 6-mercaptopurine into newly synthesized DNA to block purine synthesis enzymes. Corticosteroids, in combination with CNIs and antimetabolites, are used as the cornerstones of immunosuppressive regimens. Their immunosuppressive mechanism is diverse and may relate to interference with intracellular transcription factors and the signaling pathways of several surface receptors. Monoclonal and polyclonal antibodies may interact with cell surface antigens, such as CD3, CD20, and CD25. Immunosuppressants that are currently being developed include antibodies, FK778, Janus kinase (JAK) inhibitors, fingolimod, and blinatumomab. Among these immunosuppressive agents, those that can cause neuropsychiatric complications are CNIs, mTOR inhibitors, mycophenolate mofetil, corticosteroids, and some monoclonal antibodies, such as OKT3, belatacept, and blinatumomab.

## 4. Clinical Features Induced by Different Immunosuppressants

### 4.1. CNIs

Neurotoxicity induced by CNIs occurs at three distinct time points after transplantation: early, intermediate, and late. Most patients who use tacrolimus intravenously develop neurotoxicity on the first day after transplantation [35]. Patients who develop neurotoxicity in the intermediate or late stage demonstrate only short or intermediate survival times [36].

There are various neurological complications of CNIs, which can involve both the central nervous system (CNS) and the peripheral nervous system [37]. Mild neurological manifestations related to CNI toxicity are common and include tremors, insomnia, nightmares, sleep disturbances, headaches, vertigo, mood disturbances, and paresthesia (electric shock-like pain and severe itching) [38,39]. Serious adverse neurological effects have been relatively rarely observed and include seizures, speech disorders, cortical blindness, coma, encephalopathy, central pontine/extrapontine myelinolysis, and neuromuscular complications [14]. Tacrolimus treatment has a significantly higher incidence of neurological syndromes than CsA treatment in solid organ transplantation recipients [40,41].

Tremor is the most pronounced neurological complication associated with CNI toxicity and a fine tremor of the upper limbs can help diagnose neurological complications at early stages [13]. Tremor is significantly more common in patients treated with tacrolimus than in those treated with CsA. In a more recent trial, less than 20% of patients treated with CsA experienced tremors, while tacrolimus-related neurotoxic events occurred in up to 40% of patients [42]. In general, tremors involved both upper and lower limbs, with some patients even experiencing tremors in the head/facial muscles. Tremors exclusively involving the trunk, lower limbs, or the craniofacial area are rare in the clinic [42]. Considering that the main goal of immunosuppressive therapy is to increase the survival rates of transplant recipients, and tremors appear to be isolated with cerebellar or neuropathic involvement, this symptom tends to be ignored when its severity is not significant and does not influence the patient’s quality of life.

Seizures are common in transplant recipients undergoing CNI therapy, occurring in up to 27% of organ transplant patients [43]. Although seizures frequently occur with posterior reversible encephalopathy syndrome (PRES), the new-onset of seizures is not indicative of a poor prognosis, because most patients do well and do not require long-term antiepileptic therapy [44]. In patients with seizures, generalized tonic-clonic and occipital lobe seizures are usually observed [45]. Simple or complex partial seizures represent a localized process that may be reflected by focal electroencephalogram (EEG) abnormalities, whereas seizures that occur secondary to posterior reversible encephalopathy syndrome (PRES) frequently show short single grand mal episodes with variable theta/delta slowing [44]. Seizures associated with CNI neurotoxicity frequently originate from occipital regions [46]. PRES is a serious complication associated with immunosuppressive therapy after transplantation [47]. It is a neurotoxicity characterized by headaches, confusion, nausea and vomiting, altered mental status, visual disturbances, intracranial hemorrhage, altered sensorium, and occasionally, a focal neurological deficit [45,48,49,50,51]. In most cases, immunosuppression-associated leukoencephalopathy occurs within the first three months after transplantation and it is usually associated with intravenous treatment methods [52]. PRES appears to be significantly more common in hematopoietic or liver transplantation than in other transplantations [53].

The cranial computed tomography (CT) finding is insensitive in detecting PRES and often shows no abnormalities, while magnetic resonance imaging (MRI) has been proven to be the most sensitive imaging test. Vasogenic edema, which is a symptom of PRES, can be easily identified. Radiologists can reliably differentiate these changes from cytotoxic edema using diffusion weighted image (DWI) and apparent diffusion coefficient (ADC) maps. Moreover, the extent of abnormal T2-weighted signal intensities and DWI signal intensities correlate well with prognosis [47]. PRES predominantly affects the posterior cerebrum and the cerebral white matter, causing focal reversible vasogenic edematous changes in the specific posterior regions of the parietal and occipital lobes, which can lead to irreversible cytotoxic edema in some cases [44,54]. Grey and white matter lesions can be observed by MRI in fluid attenuated IR (FLAIR) and T2-weighted sequences, and deeper structures, such as the basal ganglia, brain stem, and deep white matter tracts, may be also affected [44,55]. Cytotoxic edema and hemorrhage are uncommon findings in these patients [44]. Typically, the characteristic of PRES is bilateral symmetric patterns of edema, usually including diffuse white matter hyperintensity with a parieto-occipital predilection [56,57]. If PRES is not diagnosed at an early stage, cerebral ischemia and massive infarction may result in an increase in morbidity and mortality [47]. Hypertension is another important symptom of PRES and, therefore, when immunosuppressants need to be continued in the clinic, blood pressure should be effectively monitored and controlled.

CPM is one of the most detrimental neurological complications after organ transplantation and the mortality due to this neurotoxicity is more than 50% [58]. The incidence of CPM is more common in liver transplantation and in patients treated with CsA than in patients treated with tacrolimus [59,60,61]. MRI features include hyperintense lesions in the center of the pons on T2 images. Rapamycin is recommended as a replacement for CNIs, because it is rarely associated with CPM. However, rapamycin is unstable and requires frequent monitoring of blood concentrations when used in clinical practice.

The number of case reports related to catatonic symptoms and akinetic mutism induced by CNI administration after organ transplantation have increased in recent years [62,63]. Even when used to treat psoriasis, CNIs have been shown to exacerbate the symptoms of paranoid schizophrenia, and then disappear a few days after discontinuation of the CNI treatment [64].

In addition to CNIs, other immunosuppressants may also manifest neuropsychiatric complications, although neurotoxicity reports are rarer for these drugs than for CNIs [37]. Mycophenolate mofetil rarely induces depression and headaches. However, seizures were frequently observed in several reports on neurological complications during rapamycin therapy. The main neurological complication of muromonab-CD3 treatment is headache, whereas patients treated with corticosteroids may experience anxiety, insomnia, mood disorders, psychotic episodes, and cognitive symptoms [65,66].

### 4.2. Antimetabolites

Methotrexate (MTX) can induce CNS toxicity that presents in the form of encephalopathy, myelopathy, or meningitis [67]. Neurological symptoms are caused by MTX are usually classified into acute, subacute, or chronic neurotoxicity. Patients who experience subacute neurotoxicity usually recover completely and spontaneously within a week, and, therefore, subsequent MTX treatment is safe for most patients [68]. Neurological symptoms induced by mycophenolate mofetil are rare and mild, manifesting as depression and headaches.

### 4.3. Corticosteroids

Neurological side effects occur in approximately 3–4% of patients who use corticosteroids [69]. Corticosteroid-induced neuropsychiatric symptoms include mood changes, behavioral disorders, and cognitive symptoms that typically manifest during the first few weeks of therapy [66]. Peripheral toxicity occurs after long-term use, usually in the form of neuromyopathy, with muscular weakness affecting the proximal and lower extremities [70]. Steroid dementia syndrome appears to be rare [71], and these symptoms may not recover completely even after the cessation of treatment [72]. Epidural lipomatosis can also induce radiculopathy due to spinal compression [73].

The adjustment or discontinuation of corticosteroids may improve some of these adverse neurological symptoms. If the psychiatric symptoms are serious, short regimens of low-dose psychotropic agents are often required (e.g., haloperidol, olanzapine, quetiapine, or risperidone).

### 4.4. Monoclonal Antibodies

Polyclonal and monoclonal antibodies are usually used to induce immunosuppression and treat graft rejection [72]. With the exception of OKT3 and belatacept, biologic agents show low incidences of adverse neurological effects. The neurotoxicities induced by OKT3 range from headaches and fever to confusion, aseptic meningitis, cerebral edema, encephalopathy, seizures, hemiparesis, nuchal rigidity, and myoclonic activity [37,74]. Furthermore, treatment with CsA after OKT3 results in an additive or synergistic adverse effect on neurological complications [75]. In general, pathological changes can be detected by a head MRI, and neurological abnormalities resolve after the cessation of the OKT3 treatment [75,76]. However, cytokine release syndrome in patients treated with OKT3 is so serious that it limits the usage of this agent. Blinatumomab, a novel recombinant murine protein, is used for the treatment of Philadelphia chromosome–negative, relapsed or refractory precursor acute lymphoblastic leukemia. There are a variety of neurological symptoms induced by blinatumomab treatment, such as somnolence, confusion, dizziness, tremor, seizure, encephalopathy, speech disorders, and loss of consciousness, which all appear to be more common in patients over 65 years of age [77].

Conditions that increase the neurotoxicity of immunosuppressant agents include pre-existing mental disorders [78], hypertension [79], electrolyte disorders including hyper and hyponatremia and hypomagnesemia [37], dysmetabolic alterations, such as hyperglycemia [37], infections that impair the function of the blood–brain barrier (BBB), hypocholesterolemia, which increases the uptake of immunosuppressant drugs in the brain [80], polymorphisms of the adenosine triphosphate (ATP)-binding cassette transporter B1 (ABCB1) gene and cytochrome pigment (CYP) gene, which decrease immunosuppressant efflux or elimination [81,82], drug interactions [82,83], a prolonged surgical period [84], and low liver function or acute liver failure [85].

## 5. Mechanisms of Neurotoxicity Induced by Different Immunosuppressants

### 5.1. CNIs

The biochemical basis of CNI-induced neurotoxicity remains unclear. It appears that high drug concentrations in the blood are correlated with neurological symptoms, but they can also occur in patients with concentrations within the therapeutic range [13,14,86]. Although both CNIs used as immunosuppressants are lipophilic, with CsA being more lipophilic than tacrolimus, they do not easily pass through the BBB [87,88]. One possible hypothesis is that tacrolimus and CsA increase the permeability of the BBB by inducing apoptosis and nitric oxide (NO) production and inhibiting P-glycoprotein (P-gp) function, which leads to further accumulation of drugs in the brain, extravasation of proteins and fluid into the interstitium, and impaired BBB function. An investigation of the effects of tacrolimus and CsA on mouse brain capillary endothelial cells (MBEC4) found that drug-treated cells experienced 1) loss of junctions with neighboring cells and detachment from the substratum, 2) chromatin condensation and fragmentation, and 3) DNA fragmentation [89]. The two drugs induced dose-independent apoptosis of the brain capillary endothelial cells, with similar effects between CsA and tacrolimus [89]. Dohgu et al. [90] reported that CsA increases NO production in brain endothelial and astroglial cells, which then participate in the impairment of BBB function. The expression of P-glycoprotein decreases with high concentrations of CNIs, leading to the inhibition of the efflux process and an enhancement of permeability. This may partly explain the mechanism of CNI-induced encephalopathy [91]. It should be mentioned that the drug concentrations in the above-mentioned studies were considerably higher than in clinical doses, and further investigation is required to determine whether normal brain capillary endothelial cells are impaired. In fact, a recent study using an in vitro BBB model, consisting of a co-culture of bovine brain capillary endothelial cells (ECs) and neonatal rat glial cells, showed that repeated exposure to 1 μM CsA, found in human plasma, had no toxic effect on BBB integrity [92]. This result was confirmed by a kinetics study, in which intracellular CsA uptake and permeability across the BBB were minimal [93]. It is also important to stress that, despite no cell damage, some key neurotransmitters, factors metabolically linked to neurotransmitters, or energy metabolism related to electrical activity that are altered at this concentration range may be responsible for the neurological disorders induced by CsA or other CNIs [94].

An alternative hypothesis is that alterations in mitochondrial function induced by CNIs contribute to neurotoxicity. A study in human umbilical endothelial cells showed that tacrolimus significantly compromised respiratory chain (RC)-complexes II and III and the mitochondrial marker enzyme, citrate synthase (CS), thus indicating a partially impaired mitochondrial function [95]. Furthermore, a similar analysis found that tacrolimus decreases oxygen consumption in human cell lines and causes a slight reduction in the synthesis of mitochondrial DNA-encoded proteins [96]. These studies suggest that the direct inhibition of the electron transport chain by CNIs, rather than effects on mitochondrial density or electron transport chain (ETC) quantity, are responsible for impaired mitochondrial function. This conclusion is contrary to an early study reporting that tacrolimus inhibits both complex III, where reactive oxygen species (ROS) are generated and complex V, where adenosine triphosphate (ATP) is depleted by ATPase activation [97]. There are two existing studies where one use glioma cells and another one uses glial cells demonstrated that tacrolimus can increase the production of ROS and decrease the antioxidant status [98,99], indicating that mitochondrial function may be impaired by tacrolimus treatment.

There is also evidence that the complex of CNIs and immunophilins may be associated with neurotoxicity. Calcineurin is expressed in several areas of the brain, including the cerebral cortex, striatum, substantia nigra, cerebellum, and hippocampus, where it regulates the dephosphorylation of Ca^2+^ channels, activity of the N-methyl-D-aspartate (NMDA) receptor, ryanodine receptor, the inositol trisphosphate (IP3) receptor, and even memory and synaptic plasticity [100,101,102]. These neurotoxic effects may depend on immune dysregulation in the nervous system, due to the pharmacologic effects of the CNI-immunophilin complex [91,103,104]. The maximal inhibitory effect of tacrolimus on calcineurin is approximately 60% (while CsA is more effective at inhibiting calcineurin) [105]. However, tacrolimus has no pharmacological effect on FK506-binding protein (FKBP) 1A (FKBP12)-null mice [106]. These findings suggest that the FK506-FKBP complex has some unknown molecular mechanisms besides its calcineurin inhibitory effect. The level of FKBP12 expression is 10–50-fold higher in the brain than in the immune system [107,108]. Tacrolimus-induced toxicity is consistent in organs with high FKBP levels, such as the brain and kidneys. Moreover, once tacrolimus enters the brain, it is eliminated slowly by binding to FKBP [109]. In an in vitro model, CsA inhibits calcineurin in the brain, even at concentrations as low as 200 nM, in a relatively short time frame. This inhibitory effect is sustained during drug administration [93]. With the exception of calcineurin inhibition by the tacrolimus-FKBP complex, the exact mechanism of neurotoxicity is not completely understood. Research on calcineurin inhibition-induced depressive-like behavior in a prefrontal cortex model raises the possibility that the blockade of the mTOR signaling pathway accounts for the neurological disorders [110]. In support of this, another study showed that receptor-associated FKBP12 participated in the intracellular mTOR activation pathway, which is well known for its critical roles in the integration of neuronal activity and synaptic inputs in multiple physiological and pathological processes [111,112]. These experimental findings are in agreement with a clinical study showing that tacrolimus induces a higher incidence of neurotoxicity than CsA [113,114].

Vasoconstriction or vascular injury [115] may also be involved in the mechanism of CNI-induced neurotoxicity. Tacrolimus may be associated with blood vessel contraction. Moreover, some investigators have suggested that, in addition to vasoconstriction caused by tacrolimus, the high infiltration pressure of the tacrolimus dissolution liquid may also affect neurotoxicity. However, although this hypothesis is interesting, it does not explain the phenomenon where patients experience CNI-induced neurologic disorders, even though their blood pressure is maintained within the normal range throughout hospitalization [63,116].

Other proposed mechanisms of CNI-induced neurotoxicity include a possible modulation of excitability properties, causing nerve membrane depolarization [117] and alterations in electrical activity [94,118], suppression of brain-derived neurotrophic factor (BDNF) and its receptor (tyrosine kinase receptor B (TrkB)), mRNA and protein expression in the hippocampus and midbrain [119], reduction of Ca^2+^ accumulation in the endoplasmic reticulum (ER) by intracellular accumulated tacrolimus [120], and significant intracellular CNI uptake, thus increasing the toxicity of other drugs administered at the same time [93]. The metabolites of CNIs may also be neurotoxic, even though they are usually not assessed in clinical practice.

### 5.2. Antimetabolites

Several biochemical pathways, including a decreased *S*-adenosylmethionine/*S*-Adenosylhomocysteine (SAM/SAH) ratio, elevated levels of homocysteine, and elevated levels of adenosine and direct toxic effects on neurons and astrocytes, may be the causes of MTX-related neurotoxicity [68].

### 5.3. Corticosteroids

Corticosteroids are often used in combination with other immunosuppressive agents. They cause neurological complications through two mechanisms that involve direct and indirect toxic effects on CNS biochemistry and electrophysiology. These include glutamate excess and neurotrophin mobilization [121] or elevated blood pressure and the vulnerability of the vasculature through the regulation of the renin-angiotensin system. Further, there are some reports suggesting that corticosteroids can make certain hippocampal and prefrontal cortical cells more vulnerable to other exogenous agents [71].

### 5.4. Monoclonal Antibodies

The mechanisms of muromonab-CD3- and belatacept-related neurotoxicity have rarely been reported. It has been postulated that cerebral complications are related to the OKT3-mediated release of cytokines [75]. This hypothesis may explain the cases of aseptic meningitis. However, it cannot explain why neurological symptoms persist after cytokine levels return to the baseline. Other studies have suggested that circulating lymphocytes and cells of the nervous system share some of the same surface antigens, such that OKT3 combines with cell surface antigens to facilitate OKT3 antibodies crossing the BBB [76]. Cytokine release syndrome induced by blinatumomab may be responsible for some of the adverse neurological effects, but further studies are warranted to clarify the precise mechanism of blinatumomab-induced neurotoxicity [77].

## 6. Management

Immunosuppressants, particularly CNIs, can induce neurotoxicity in solid organ transplantation cases. The management of blood concentrations of the drugs by therapeutic drug monitoring, individual therapy based on pharmacogenetics, and the early recognition of symptoms using electrophysiological and imaging strategies, may help avoid neurotoxicity [82,122,123]. However, even with these measures in place, the incidence of neurotoxic symptoms remains high (3–32%) [79,124,125]. Once neurotoxicity occurs, reducing the dosage of the drug, switching from tacrolimus to CsA or vice versa, or using an alternative immunosuppressant agent, such as mycophenolate mofetil, have proven to be effective approaches to reverse this neurotoxicity [63,126,127]. A study comparing the effects of tacrolimus and rapamycin on bioelectrical activity and evoked field excitatory postsynaptic potential (fEPSP) in the CA1 area of hippocampal tissues has also suggested that rapamycin could replace CNIs in the event of seizures [128]. However, in some cases, switching between tacrolimus and CsA has not been effective at improving neurotoxicity. Moreover, the continued administration of CNIs, in combination with drugs that treat, neuropsychiatric manifestations may be considered the best approach, given that considering that switching immunosuppressants may elevate the risk of graft rejection in some patients and reducing the CNI dosage does not always improve the symptoms. For example, olanzapine, coupled with the continued use of tacrolimus, has been shown to resolve manic episodes [129]. Olanzapine has also been considered for the treatment of catatonic mutism after liver transplantation [130]. Benzodiazepines can improve catatonia, especially akinetic–hypokinetic catatonic syndromes [40,131]. The neurotoxicity associated with CNIs is strongly correlated with the intracerebral concentration of the drugs [132]. Sakamoto et al. showed that the continuous administration of tacrolimus is more advantageous than intermittent administration to reduce neurotoxicity in rats [133]. In addition, tacrolimus-induced neurotoxicity and nephrotoxicity can be ameliorated, while maintaining its immunosuppressive effects, by treating rats in the dark phase [134]. According to a case report, red-blood cell exchange improved the clinical status of a 60-year-old woman with severe neurological impairment due to tacrolimus overexposure. Hence, red-blood cell exchange may be an effective therapy to reduce tacrolimus neurotoxicity [135]. This blood purification therapy has also been shown to be effective in ifosfamide-induced severe concurrent neurotoxicity and nephrotoxicity [136].

## 7. Neuroprotective Effects

### 7.1. CNIs

The immunosuppressants—tacrolimus and CsA—have neuroprotective effects in animal models of focal and global cerebral ischemia [137,138,139], portacaval anastomosis and hyperammonemia [140], intracerebroventricular streptozotocin-induced neurotoxicity [141], and temporal lobe epilepsy (the turnover of tacrolimus is much faster in rats than in humans) [142].

When wild-type mice are treated with tacrolimus for one week, their neocortices show longer total dendritic arbors and more complex branching further away from the cell body, compared to untreated animals [143]. There is some experimental data to indicate that the neuroprotective effects induced by tacrolimus and CsA may be related to calcineurin inhibition, NFκB activation [144], downregulation of proinflammatory/cytotoxic cytokines [145], decreased NO synthetase-mediated NO production [137,140,146], inhibition of Ca^2+^ release by both the ER and mitochondria, as well as mitochondrial permeability transition (mPT) (CsA) [147], deceased apoptosis and c-jun protein expression in neurons [148], calcineurin-independent mechanisms [149], activation of pro-survival pathways by BDNF and its receptor, tropomyosin receptor kinase A (TrkA) [150], and excitotoxic neuronal death [151].

However, although the neuroprotective functions of tacrolimus have been demonstrated in various nerve injury models, these functions have been challenged by models of inherited peripheral myelinopathies treated with tacrolimus. For example, tacrolimus exacerbates neurological abnormalities, including demyelination and dysmyelination-associated axon loss in inherited de/dysmyelination mice, while the peripheral nerves of wild-type mice do not show any neurotoxic symptoms after treatment with tacrolimus [152].

Interestingly, a recent study found that tacrolimus and CsA treatment had no better long-term effects than treatment with the vehicle alone (cremophor and ethanol mixture). Moreover, the drug-treated group showed even more significant decreases in brain weight. Therefore, Setkowicz and Guzik [153] concluded that the neuroprotective effects observed in rat brains injured mechanically at the early developmental stages may result from the influence of the vehicle alone. Further studies and more investigations are needed to clarify the potential neuroprotective effects and mechanisms of CNIs.

### 7.2. mTOR Inhibitors

mTOR is associated with the pathogenesis of neurological, cognitive, and psychiatric disorders, such as epilepsy, stroke, traumatic brain injury, parkinsonism, spinal cord injury, and Alzheimer’s disease [154]. In a mouse model of epilepsy induced by knocking-out the protein phosphatase and tensin homolog (PTEN), mTOR activity increases in neurons. Therefore, reducing mTOR activity may effectively suppress epileptogenesis and alleviate the symptoms of this disease [155]. The role of mTOR in cerebral ischemia has also been reported in some rodent experiments. Some studies have shown that the mTOR pathway has neurotoxic effects, while others have reported the opposite finding that the mTOR pathway has neuroprotective effects. Some reports have suggested that suppressing the pharmacological effects of the mTOR pathway can regulate autophagy and result in neuroprotection, whereas other reports have suggested that the neurotoxicity of mTOR inhibitors is related to the promotion of autophagic processes, long-term activation of Akt, and activation of S6 kinase 1 (S6K1) occurring in brain cells after a stroke [154,156,157,158]. The study by Chen and co-workers [159] may explain the paradoxical effects of the mTOR inhibitor, rapamycin. In that study, rapamycin was shown to cause a paradoxical, but transient, increase in mTOR pathway activation in a kainite injection model, and in normal rats, by increasing the phosphorylation of S6. These results suggest that the effects of rapamycin on mTOR are related to the type or period of stimuli and the dose administered [160].

## 8. Conclusions

Neurological disorders are common after solid organ transplantation. The reasons for these neurotoxicities are multifactorial, ranging from the effects of immunosuppressive agents to pre-transplantation disease. In this article, we discussed the neurological complications resulting from immunosuppressive therapy in five categories: the process of alloimmune responses, the classification of immunosuppressive agents, their clinical features, their mechanisms, and their clinical management. Interestingly, some studies have shown that these immunosuppressive agents may have neuroprotective effects. However, two recent studies have reported contradictory findings, suggesting that further studies are required to clarify the potential neuroprotective effects of immunosuppressive agents.

## Figures and Tables

**Table 1:** The details of various immunosuppressive agents shown according to their classification.

Corticosteroids	
Generic Name	Prednisone; Prednisolone; Methylprednisolone; Dexamethasone
Trade Name	Prelone^®^, Orapred^®^, Millipred^®^, Orapred ODT^®^; Prednisol^®^, Pred Forte^®^, Pred Mild^®^, Omnipred^®^; Medrol^®^, Medrol Dosepak^®^, MethylPREDNISolone Dose Pack^®^, Solu-Medrol^®^; Decadron^®^, Dexamethasone Intensol^®^, Dexasone^®^, Hexadrol^®^
Mechanism of Action	The mechanisms of action are diverse and include interference with intracellular transcription factors and signaling pathways of several surface receptors, including the T cell antigen receptor and downstream kinases, thereby blocking the transcription of cytokine genes and inhibiting cytokine production by T cells and macrophages [32].
Role in Therapy	Maintenance; high doses of corticosteroids (>1 mg/kg), used for induction therapy in transplantation; treatment of acute cellular rejection and AMR [3,32].
Adverse Effects	Hypertension, hyperlipidemia, glucose intolerance, malignancy, Cushingoid features, sleep disturbances, mood changes, impaired wound healing, osteoporosis, psychosis, photosensitivity, acne hirsutism, avascular necrosis, weight gain, fluid retention, increased appetite, menstrual irregularities, growth inhibition, GI disturbance, cataracts, infection [3].
Monitoring Parameters	Glucose, blood pressure, fasting lipid panel, weight, DEXA scan, eye exam, intensive organ function monitoring [31].
Other Information	Their role in the maintenance of immunosuppression is under investigation because of severe side effects during long-term use, but an immunosuppressive strategy without steroids could be only tried in low immunological risk transplant recipients; it also seems that treatment of steroids 1 h prior to ATG preoperatively may minimize CRS [3,4].
Purine synthesis inhibitors	
Generic Name	Azathioprine; Mycophenolate mofetil; Mycophenolate sodium; Cyclophosphamide
Trade Name	Imuran^®^; Cellcept^®^; Myfortic^®^; Cytoxan^®^, Neosar^®^, Endoxan^®^
Mechanism of Action	Two distinct mechanisms participate in the inhibition of de novo DNA synthesis block cell division and then block cell division. AZA is a prodrug for 6-mercaptopurine, Mycophenolate mofetil is a prodrug of MPA and Mycophenolate sodium is an enteric-coated formulation of MPA. AZA blocks purine synthesis enzymes by incorporating into newly synthetized DNA and, finally, impedes DNA and RNA synthesis [31]. MPA selectively and noncompetitively inhibits a key enzyme in the de novo synthesis of purine named IMPDH and thus, inhibits proliferation of T and B lymphocyte [32].
Role in Therapy	Maintenance
Adverse Effects	AZA: Hepatotoxicity, bone marrow suppression, malignancies (high dosages), macrocytic anemia, GI disturbance, alopecia, pancreatitis, infections [31]; MMF and Mycophenolate sodium: Dyslipidemia, DM, infections, bone marrow suppression, GI symptoms and anemia are common, while nephrotoxicity, neurotoxicity, and hepatotoxicity are uncommon [18,30]; CP: Low blood count, alopecia, GI symptoms, poor appetite, discoloration of the skin or nails.
Monitoring Parameters	AZA: CBC, LFT, amylase, lipase, TPMT enzyme level; MMF and Mycophenolate sodium: CBC, REMS; CP: CBC, LFT, KFT [31].
Other Information	Newer trials have shown that AZA and MMF have similar efficacy. Low or absent TPMT activity is associated with increased AZA-associated myelosuppression. MPA is associated with pregnancy loss and congenital malformations when used during pregnancy. MPA may be of special interest in preventing the rise of DSA titers in pre-sensitized recipients. Patients with renal dysfunction need dosage adjustment when using MPA [28,31]. CP is associated with pregnancy loss and congenital malformations when used during pregnancy.
CNIs	
Generic Name	Tacrolimus; Cyclosporine
Trade Name	Prograf^®^, Graceptor^®^, Advagraf^®^, Envarsus XR^®^, Astagraf XL^®^; Neoral^®^, Gengraf^®^, Sandimmune^®^
Mechanism of Action	CNIs block signal transduction by activated NFAT through two distinct mechanisms. Tacrolimus binds to FKBP12 while CsA in combination of cyclophilin inhibits calcineurin-mediated dephosphorylation of NFAT, ultimately preventing cytokine transduction including IL-2 and IFNγ and T cell activation. In humoral immune response, CNIs interfere with T helper signals rather than targeting B cell directly [32].
Role in Therapy	Maintenance
Adverse Effects	Often dose- and concentration- dependent, nephrotoxicity, infections, hyperkalemia, hypomagnesemia, hyperuricemia, cholelithiasis, GI symptoms, malignancy; tacrolimus > CsA: insulin-dependent diabetes mellitus, neurotoxicity; CsA > tacrolimus: hypertension, hypercholesterolemia, hyperlipidemia; CsA only: gingival hyperplasia, hirsutism; tacrolimus only: alopecia [3,4,31].
Monitoring Parameters	Trough levels, serum creatinine, potassium, magnesium, uric acid [31]
Other Information	Tacrolimus seems more effective than CsA-based immunosuppressive regimens, so tacrolimus-based immunosuppression usually used as a first-line therapy after transplantation. Tacrolimus is metabolized by CYP3A and has potential drug interactions. Neurotoxicity more likely occurs in liver transplant patients with low serum cholesterol levels. Patients with hepatic dysfunction or advanced age have high risk of drug interactions after CSA [3,4,18,31].
mTOR inhibitors	
Generic Name	Sirolimus (Rapamycin); Everolimus;
Trade Name	Rapamune^®^; Certican^®^, Zortress^®^
Mechanism of Action	These drugs in combination of FKBP12 inhibit mTOR and impede the translation of mRNA-encoding proteins which are necessary to the cell cycle, thus reducing IL-2-mediated T cell proliferation and cytokine production. In contrast to CNIs, they seem to do not influence the early phase of T-cell activation [31,32].
Role in Therapy	Maintenance
Adverse Effects	Dyslipidemia, mucositis, edema, proteinuria, wound-related reactions, mouth ulcers, bone pain, diarrhea, pneumonitis, venous thromboembolism, infections, low blood count [3]
Monitoring Parameters	Trough levels, fasting lipid panel, CBC, LFT [31]
Other Information	Only sirolimus is reported to have direct inhibitory effects on the proliferation of B cells and their differentiation into plasma cells [32]. An mTOR inhibitor–based regimen is under investigation for low risk of nephrotoxicity or neurotoxicity when used alone [3].
Monoclonal antibodies	
Generic Name	Muromonab-CD3; Rituximab; Basiliximab; Daclizumab; Alemtuzumab; Eculizumab
Trade Name	Orthoclone OKT3^®^; Rituxan^®^; Simulect^®^; Zinbryta^®^; Campath^®^, Lemtrada^®^; Soliris^®^
Mechanism of Action	Muromonab-CD3: first monoclonal antibody approved for use in solid-organ transplantation, direct against the CD3 marker on all mature human T cells [30]. Rituximab: a murine/human chimeric monoclonal antibody directly targets the CD20 surface marker on B cells [33]. Basiliximab: a murine/human chimeric monoclonal antibody competitively inhibits CD25 complex, the alpha subunit of the IL-2 receptor which present only on activated and non-resting T cell, thereby inhibiting T cell proliferation [34]. Daclizumab: a humanized monoclonal antibody similar to Basiliximab, has high specificity and affinity against CD25 complex [34]. Alemtuzumab: a recombinant DNA-derived, humanized anti-CD52 monoclonal antibody targets T and B lymphocytes, NK cells, monocytes, and macrophages, finally leading to rapid and powerful depletion of T and B lymphocytes, and monocytes [31]. Eculizumab: a humanized monoclonal antibody binds to complement C5 with high affinity and blocks complement cascade by preventing the formation of the terminal membrane attack complex [34].
Role in Therapy	Muromonab-CD3: withdrawn Rituximab: Desensitization, treatment of AMR, and for cases of PTLD [30,31] Basiliximab, Daclizumab: Induction Alemtuzumab: Induction, treatment of AMR and steroid-resistant rejection [31] Eculizumab: Desensitization, treatment of AMR [3]
Adverse Effects	Muromonab-CD3: Serious CRS Rituximab: Bone marrow suppression, infusion-related events [3] Basiliximab: Rare; infections, bone marrow suppression, hypersensitivity reactions [3] Daclizumab: GI disturbance, rare lymphoproliferative disorders and malignancies [33] Alemtuzumab: Bone marrow suppression, infusion reaction, infections, mild CRS, headaches, induction of autoimmune disease, a possible increased risk of PTLD [31,34] Eculizumab: Increased risk for gram-negative bacterial infection, bone marrow suppression [3,30]
Monitoring Parameters	Alemtuzumab: Vital signs, CBC, absolute lymphocyte count [31]
Other Information	Rituximab: Has been tested as an induction agent in cell therapy [3]. Basiliximab: Induction therapy using basiliximab has higher rejection rates [3]. Alemtuzumab: Usage in induction and acute rejection treatment is still under study; has a similar immunosuppressive effect to ATG, but less side effects. Pre-treatment of diphenhydramine and acetaminophen can decrease side effects [30,31]. Eculizumab: The usage for immunosuppressants has been only reported in case reports and observational studies, has limited efficacy and high cost [34].
Polyclonal antibodies	
Generic Name	Antithymocyte globulin
Trade Name	Thymoglobulin^®^
Mechanism of Action	This drug depletes the number of circulating T lymphocytes by antibody–dependent cell–mediated or complement-depend cytotoxicity and their interaction with T cell surface antigens, may result in apoptosis, which alters T cell activation and homing [32].
Role in Therapy	Induction; treatment of steroid-resistant rejection [3]
Adverse Effects	Malignancies, infections, bone marrow suppression, CRS, pulmonary edema, phlebitis, pruritis, erythema, serum sickness [3,31]
Monitoring Parameters	White blood cells, platelet count, vital signs, CD3 count [31]
Other Information	To prevent an intense CRS, pre-treatment with systemic glucocorticoids, antihistamines and antipyretics should precede drug administration; preferred in sensitized patients without DSAs [31,32].
Co-stimulation blockade agent	
Generic Name	Belatacept
Trade Name	Nulojix^®^
Mechanism of Action	An agent mimics soluble CTLA-4 and binds to CD86/80 on APCs, thus blocking T-cell activation. Moreover, it maybe indirectly prevent production of antigen-specific antibody (IgG, IgM, and IgA) by B lymphocytes or directly affect B lymphocytes [30,32].
Role in Therapy	Induction; maintenance
Adverse Effects	Malignancies, bone marrow suppression, diarrhea, infection, edema, hypertension, dyslipidemia, DM, proteinuria, electrolyte disorders, dyspnea, purpura, transaminitis, temporal lobe epilepsy. More than 20% of patients experience side effects [31,34].
Monitoring Parameters	EBV serostatus (prior to treatment) [31]
Other Information	Only used for adult patients; no drug-drug interactions; patients with renal or hepatic impairment need no dosage adjustment; contraindicated in recipients who are EBV seronegative or with unknown EBV serostatus [4,30,31].
Immunosuppressants in development	
Generic Name	FK778; Tofacitinib (CP-690550); Bortezomib (PS341); Tocilizumab; IdeS (imlifidase); Fingolimod (FTY720); Alefacept; ASKP1240; Voclosporin (ISA247); Sotrastaurin (AEB071); Siplizumab; TOL101; Efalizumab; Belimumab; Sutimlimab (BIVV009); C1-INH (C1 esterase inhibitor)
Trade Name	none; Xeljanz^®^; Velcade^®^; Actemra^®^; none; Gilenya^®^; Amevive^®^; none; Luveniq^®^; none; none; none; Raptiva^®^, Genentech^®^, Merck Serono^®^; Benlysta^®^; none; Berinert^®^, Cinryze^®^, Haegarda^®^
Mechanism of Action	FK778: An agent blocks pyrimidine synthesis by blockade of DHODH and inhibition of tyrosine kinase activity, thus inhibiting both T-cell and B-cell function; moreover, it can directly inhibit lymphocyte activation, attenuate lymphocyte-endothelium interactions [4,28]. Tofacitinib: A JAK3 inhibitor, that exerts its effects by uncoupling cytokine receptor signaling from downstream STAT transcriptional activation and subsequently, suppressing various cytokine-regulated signaling, thus influencing lymphocyte activation, proliferation, differentiation, and function [4,29,33]. Bortezomib: A reversible 26S proteasome inhibitor that can delete non-transformed plasma cells, which is critical to alloantibodies [30]. Tocilizumab: A first-in-class, humanized, monoclonal antibody directed against IL-6R [29,33]. IdeS: An enzyme from Streptococcus pyogenes that specifically cleaves human IgG antibodies [33]. Fingolimod: A structural analogue of sphingosine, metabolized by sphingosine kinases to fingolimod-phosphate in the cell; this active metabolite can entrap lymphocytes in secondary lymphoid organs and reduce their number in peripheral blood, thus reducing cell-mediated immune responses [4,28]. Alefacept: Directed against the extracellular CD2 receptor expressed on T lymphocytes thus inhibiting lymphocyte activation and production; blocks the CD2/LFA-3 interaction and impedes helper T-cell adhesion to APCs [29,34]. ASKP1240: A novel, fully human anti-CD40 monoclonal antibody, is currently under study in phase II clinical trials in kidney transplantation [29]. Voclosporin: A novel oral semisynthetic analogue of CsA, inhibits calcineurin [29]. Sotrastaurin: An oral protein kinase C inhibitor that can block T-cell activation [29]. Siplizumab: A novel humanized monoclonal antibody, binds to CD2 antigen on T lymphocyte or NK cell [29]. TOL101: A highly selective murine monoclonal antibody targeting the αβ-TCR [29]. Efalizumab: An anti-lymphocyte function-associated antigen molecule that inhibits lymphocyte activation and migration [29]. Belimumab: A human monoclonal antibody that inhibits BAFF [29]. Sutimlimab: Selectively blocks the classical pathway of complement -specific serine protease C1s to prevent the formation of the classic C3 convertase pathway [29]. C1-INH: A serine-protease inhibitor inhibits complement system by binding to and inactivating C1r and C1s and dissociating the C1 complex [29].
Role in THERAPY	FK778: Further development for the treatment of transplantation has been discontinued [4,28]. Tofacitinib: Withdrawn in transplantation. Bortezomib: Desensitization, treatment of AMR [30]. Tocilizumab: Desensitization [29] IdeS: Desensitization [33] Fingolimod: No further development for the treatment of transplantation [4,28]. Alefacept: Withdrawn in transplantation. ASKP1240: Immunosuppressive effects in nonhuman primates have been proven [29]. Voclosporin: Its efficacy in preventing acute rejection is as potent as tacrolimus by a phase 2b PROMISE study [29]. Sotrastaurin: May be an alternative therapy for Cis [29]. Siplizumab: Has been tested as an induction drug in a human study [29]. TOL101: Has been tested as an induction agent to prevent rejection is currently under study in phase II clinical trials [29]. Efalizumab: Withdrawn. Belimumab: The usage as supplement to standard-of-care immunosuppressive therapy in renal transplantation has been proven by a phase II study [29]. Sutimlimab: A single-arm pilot trial showed that BIVV009 effectively blocks the alloantibody-triggered classical pathway activation in kidney transplant recipients [29]. C1-INH: The results of a recent placebo-controlled trial suggested that C1-INH replacement may be useful in the treatment of AMR [29].
Adverse Effects	Tofacitinib: Infection, CMV disease, PTLD, anemia, neutropenia [29]. Bortezomib: GI syndromes, asthenia, neurotoxicity, bone marrow suppression, shingles [3,30]. Tocilizumab: Infections. Fingolimod: Bradycardia, macular oedema, increased airway resistance, a “first-dose” negative chronotropic effect [4,28]. Alefacept: Malignancies. Sotrastaurin: GI events are common [29]. Efalizumab: Infections, PML, PTLD. Belimumab: Infection, hypersensitivity, malignancy.
Monitoring Parameters	Tofacitinib: Drug serum levels [4,29,33].
Other Information	FK778: There have been no results proving the efficacy of FK778 in phase III studies. Therefore, its development was been discontinued for organ transplantation in 2006 [29]. Tofacitinib: When combined with MMF, the rates of viral infection and viral-associated malignancies may increase [29]. Bortezomib: Small, non-randomised trials suggest efficacy in AMR; may decrease AMR in highly sensitised individuals [30]. IdeS: IdeS has been proven to effectively reduce anti-HLA antibody levels in highly sensitized patients by a phase II study; clinical trials in sensitized kidney patients are ongoing [29]. Fingolimod: It is now approved for use in MS, but its mechanism is still unknown. Alefacept: Its use for the prevention of graft-versus host disease is under investigation. ASKP1240: Further clinical III studies are needed [29]. Voclosporin: Low-dose voclosporin may reduce incidence of new-onset diabetes after transplantation [29]. Sotrastaurin: High-dose sotrastaurin may be associated with faster heart rates [29]. Sutimlimab: Undergoing phase clinical III trail [29]. C1-INH: Further studies are needed to confirm the safety and efficacy of C1-INH in the treatment of AMR [29].

Abbreviations: AMR, antibody-mediated rejection; APC, antigen-presenting cell; ATG, anti-thymocyte globulin; AZA, azathioprine; BAFF, B-cell activating factor; CBC, complete blood count; CD, cluster of differentiation; CNIs, calcineurin Inhibitors; CP, cyclophosphamide; CRS, cytokine release syndrome; CsA, cyclosporine; CTLA4, cytotoxic T lymphocyte–associated antigen 4; CYP3A4, cytochrome P3A4; C1-INH, C1 esterase inhibitor; DEXA, dual-energy X-ray absorptiometry; DHODH, dihydroorotic acid dehydrogenase; DM, diabetes mellitus; DSA, donor-specific antibodies; EBV, Epstein-Barr virus; FKBP, FK506-binding protein; GI, gastrointestinal; HUS/TMA, hemolytic uremic syndrome/thrombotic microangiopathy; Ides, immunoglobulin G-degrading enzyme derived from Streptococcus pyogenes; IFN, interferon; IL-2, interleukin-2; IL-6R, IL-6 receptor; IMPDH, inosine-5’-monophosphate dehydrogenase; JAK, janus kinase; KFT, kidney function test; LFT, liver function test; MHC, major histocompatibility complex; MMF, mycophenolate mofetil; MPA, mycophenolic acid; MS, multiple sclerosis; mTOR, mammalian target of rapamycin; muromonab-CD3, mouse monoclonal immunoglobulin G2 antibody to cluster of differentiation 3; NFAT, nuclear factor of activated T-cells; PML, progressive multifocal leukoencephalopathy; PTLD, post-transplant lymphoproliferative disorder; REMS, pregnancy test in women of childbearing age; STAT, signal transducers and activators of transcription; TPMT, thiopurine methyltransferase; TCR, T cell receptor.

## Abbreviations

ABCB1	ATP-binding cassette transporter B1
ADC	Apparent diffusion coefficient
APC	Antigen presenting cell
ATP	Adenosine triphosphate
BBB	Blood-brain barrier
BCR	B-cell receptor
BDNF	Brain-derived neurotrophic factor
CD	Cluster of differentiation
CNI	Calcineurin inhibitor
CNS	Central nervous system
CPM	Central pontine myelinolysis
CS	Citrate synthase
CsA	Cyclosporin A
CT	Computed tomography
CYP	Cytochrome pigment
DWI	Diffusion weighted image
EC	Endothelial cell
EEG	Electro-encephalogram
ER	Endoplasmic reticulum
ETC	Electron transport chain
fEPSP	Field excitatory postsynaptic potentials
FKBP	FK506-binding protein
FLAIR	Fluid-attenuated IR
IFN-γ	Interferon-gamma
IL-2	Interleukin-2
IMPDH	Inosine-5′-monophosphate dehydrogenase
IP3	Inositol trisphosphate
JAK	Janus kinase
MAP	Mitogen activated protein
MBEC4	Mouse brain capillary endothelial cells
MHC	Major histocompatibility complex
mPT	Mitochondrial permeability transition
MRI	Magnetic resonance imaging
mTOR	Mammalian target of rapamycin
MTX	Methotrexate
muromonab-CD3	Mouse monoclonal immunoglobulin G2 antibody to cluster of differentiation 3
NFAT	Nuclear factor of activated T cells
NFκB	Nuclear factor kappa B
NMDA	N-methyl-D-aspartate
NO	Nitric oxide
P-gp	P-glycoprotein
PRES	Posterior reversible encephalopathy syndrome
PTEN	Phosphatase and tensin homolog
RAS	Renin–angiotensin system
RC	Respiratory chain
ROS	Reactive oxygen species
SAH	S-adenosylhomocysteine
SAM	S-adenosylmethionine
S6K1	S6 kinase 1
TCR	T-cell receptor
Tfh	T follicular helper
TNF-α	Tumor necrosis factor-alpha
TrkA	Tropomyosin receptor kinase A
TrkB	Tyrosine kinase receptor B
